# Azilsartan Reduced TNF-α and IL-1β Levels, Increased IL-10 Levels and Upregulated VEGF, FGF, KGF, and TGF-α in an Oral Mucositis Model

**DOI:** 10.1371/journal.pone.0116799

**Published:** 2015-02-17

**Authors:** Aurigena Antunes de Araújo, Hugo Varela, Caroline Addison Carvalho Xavier de Medeiros, Gerly Anne de Castro Brito, Kênio Costa de Lima, Ligia Moreno de Moura, Raimundo Fernandes de Araújo

**Affiliations:** 1 Postgraduate Programs in Public Health and Pharmaceutical Science, Department of Biophysics and Pharmacology, Federal University of Rio Grande Norte (UFRN), Natal, RN, Brazil; 2 Postgraduate Program in Public Health, UFRN, Natal, RN, Brazil; 3 Department of Biophysics and Pharmacology, UFRN; and Postgraduate Program in Health and Society, State University of Rio Grande Norte (UERN), Natal, RN, Brazil; 4 Postgraduate Program in Pharmacology and Morphology, Department of Morphology, Federal University of Ceará (UFC), Fortaleza, CE, Brazil; 5 Postgraduate Program in Public Health and Health Science, Department of Dentistry, UFRN, Natal, RN, Brazil; 6 Postgraduate Program in Public Health, UFRN; and University Potiguar (UnP), Natal, RN, Brazil; 7 Postgraduate Program in Functional & Structural Biology and Health Science, Department of Morphology, UFRN, Natal, RN, Brazil; Zhongshan Hospital Fudan University, CHINA

## Abstract

Oral mucositis (OM) is a common complication of treatments for head and neck cancer, particularly radiotherapy with or without chemotherapy. OM is characterised by oral erythema, ulceration, and pain. The aim of this study was to evaluate the effect of azilsartan (AZT), an angiotensin II receptor antagonist, on 5-fluorouracil (5-FU)-induced oral mucositis (OM) in Syrian hamsters. OM was induced by the intraperitoneal administration of 5-FU on experimental days 1 (60mg/Kg) and 2 (40mg/Kg). Animals were pretreated with oral AZT (1, 5, or 10 mg/kg) or vehicle 30 min before 5-FU injection and daily until day 10. Experimental treatment protocols were approved by the Animal Ethics Committee Use/CEUA (Number 28/2012) of the UFRN. Macroscopic analysis and cheek pouch samples were removed for histopathologic analysis. Myeloperoxidase (MPO), Malonyldialdehyde (MDA), interleukin-1 beta (IL-1β), interleukin-10 (IL-10), and tumour necrosis factor-alpha (TNF-α) were analysed by Enzyme Linked Immuno Sorbent Assay (ELISA). Vascular endothelial growth factor (VEGF), fibroblast growth factor (FGF), keratinocyte growth factor (KGF), and transforming growth factor (TGF)-α were measured by immunohistochemistry. Analysis of variance followed by Bonferroni’s test was used to calculate the means of intergroup differences (p ≤ 0.05). Treatment with 1 mg/kg AZT reduced levels MPO (p<0.01), MDA (p<0.5) and histological inflammatory cell infiltration, and increased the presence of granulation tissue. AZT treatment at 1 mg/kg reduced the TNF-α (p<0.05) and IL-1β (p<0.05) levels, increased the cheek pouch levels of IL-10 (p<0.01), and upregulated VEGF, FGF, KGF, and TGF-α. Administration of AZT at higher doses (5 and 10 mg/kg) did not significantly reverse the OM. AZT at a dose of 1 mg/kg prevented the mucosal damage and inflammation associated with 5-FU-induced OM, increasing granulation and tissue repair.

## Introduction

Oral mucositis (OM) is a common complication of treatments for head and neck cancer, particularly radiotherapy with or without chemotherapy. OM is characterised by oral erythema, ulceration, and pain. The condition can predispose patients with neutropenia to septicaemia [1,2].

There are five phases in OM pathogenesis. The initiation phase involves the initial injury to cells by radiotherapy and/or chemotherapy. This injury may be induced directly via DNA damage or (more commonly) indirectly via reactive oxygen species. The consequent activation of various enzymes and transcription factors eventually leads to the upregulation of genes coding for inflammatory cytokines, such as tumour necrosis factor (TNF)-α, interleukin (IL)-1β, and IL-6, which target the submucosa and basal epithelium. The resulting inflammation and tissue damage lead to ulceration and subsequent bacterial colonisation, further feeding a vicious cycle of inflammatory cytokine-mediated damage. The final healing phase involves signalling via the extracellular matrix, resulting in epithelial proliferation, epithelialisation, and reestablishment of the mucosal barrier [3].

Different therapeutic approaches for cancer treatment-induced OM have been reported, including intensive oral hygiene care [4], antimicrobial agents [5], anti-inflammatory agents [6], cytokines, growth factors [7], and topical agents, such as laser therapy [8,9] or medicinal plants [10,11]. To date, however, no single intervention has been able to prevent and treat OM; combinations of treatments acting on different phases of OM must be used. Moreover, it is still unclear which strategies reduce OM, as no evidence supports any treatment as having superior efficiency and efficacy [12].

The mucosal immune response, including tolerogenic prevention of inflammatory reactions and the secretion of antigen-nonspecific suppressor cytokines (e.g., IL-10) [13]. Various signalling pathways have the ability to increase keratinocyte migration and proliferation. These pathways include epidermal growth factor (EGF) family members, such as transforming growth factor-alpha (TGF-α) [14]. In addition, other growth factors are involved in granulation tissue formation, such as vascular endothelial growth factor (VEGF) [15] and fibroblast growth factor (FGF)-2 [16].

Our group has studied angiotensin receptor blockers (ARBs) because these drugs have been shown to interfere with pathways that mediate inflammation in an experimental animal model [17–20]. The purpose of the study reported in this paper was to investigate the anti-inflammatory activity of azilsartan (AZT) in an experimental model of OM.

## Material and Methods

### Animals

Male adult Syrian hamsters weighing 150 to 200 g were obtained from the vivarium of the Department of Biophysics and Pharmacology of the Federal University of Rio Grande Norte (UFRN) and Potiguar University (UNP), Brazil. Experimental and animal treatment protocols were approved by the Animal Ethics Committee/CEUA of the UFRN (no. 28/2012). All animals were housed in an animal room under standard laboratory conditions, at 22 ± 2°C with a 12-h/12-h light/dark cycle. Animals were fed pelleted food and water *ad libitum*. They were acclimatised for 7 days and fasted for 12 h before the experiments. All efforts were made to minimize the number of animals used and their suffering degree.

### Induction of experimental OM

Hamsters were divided into six groups (n = 6/group). On days 1 and 2 of the experiment, 5-FU was administered intraperitoneally (i.p.) at 60 and 40 mg/kg, respectively, in accordance with an experimental OM model described by Medeiros *et al*. [21]. On day 4, animals were anaesthetised with 2% xylazine (10 mg/kg) and 10% ketamine (70 mg/kg). The cheek pouch mucosa was irritated by superficial scratching to potentiate OM. Irritation was performed twice, by dragging the tip of an 18-gauge needle across the everted cheek pouch in a linear manner, to reproduce the clinical signs of chronic irritation and to create a condition favourable for OM by mechanical trauma (MT). Animals were sacrificiedon day 10 by an overdose of anaesthesia with 2% thiopental (80 mg/kg, i.p.). After death a cardiac puncture was performed.

### Experimental groups

Hamsters in the three experimental OM groups were treated with oral AZT (EDARBI, USA) at a dosage of 1, 5, or 10 mg/kg (groups AZT1/5-FU, AZT5/5-FU, and AZT10/5-FU, respectively). Oral AZT was administered 30 min before 5-FU injection, and then daily until the hamsters were sacrificied on day 10. Hamsters in the three control groups (Normal, MT, and 5-FU/Saline groups) were treated with saline instead of AZT, with or without 5-FU and/or MT treatment. The Normal group was not treated with 5-FU or MT; the MT group was not treated with 5-FU, but received MT on day 4; and the 5-FU/Saline group received i.p. 5-FU on days 1 and 2 (60 and 40 mg/kg, respectively), and MT of the cheek pouches on day 4.

### Macroscopic analysis of cheek pouch

Photographs were used for scoring lesions. For macroscopic analysis, inflammatory aspects, such as erythema, erosion, vasodilatation, epithelial ulcerations, and abscesses, were evaluated in a single-blind fashion and graded as follows[21]: Score 0, completely healthy cheek pouch with no erosion or vasodilatation; Score 1, presence of erythema, but no evidence of erosion in the cheek pouch; Score 2, severe erythema, vasodilation, and surface erosion; Score 3, formation of ulcers in one or more faces of the mucosa, but not affecting more than 25% of the surface area of the cheek pouch, as well as severe erythema and vasodilatation; Score 4, cumulative formation of ulcers of about 50% of the surface area of the cheek pouch; and Score 5, virtually complete ulceration of the cheek pouch mucosa, in which the fibrosis makes oral mucosa exposure difficult.

### Histopathologic analysis

Specimens were fixed in 10% neutral buffered formalin, dehydrated, and embedded in paraffin. Sections measuring 5-μm thick were obtained for haematoxylin-eosin staining and were examined by light microscopy (×40 magnification). Inflammatory cell infiltration, vasodilatation, presence of haemorrhagic areas, oedema, ulcerations, and abscesses were determined in a single-blinded fashion and graded as follows [21]: Score 1, normal epithelium and connective tissue without vasodilatation, absence of or discreet cellular infiltration, and absence of haemorrhagic areas, ulcerations, or abscesses; Score 2, discreet vasodilatation or re-epithelisation areas, discreet inflammatory infiltration with mononuclear prevalence, and absence of haemorrhagic areas, oedema, ulcerations, or abscesses; Score 3, moderate vasodilatation, areas of hydropic epithelial degeneration, inflammatory infiltration with neutrophil prevalence, presence of haemorrhagic areas, oedema, and eventual ulceration, and absence of abscesses; and Score 4, severe vasodilatation, and inflammatory infiltration with neutrophil.

### Immunohistochemical analysis of VEGF, FGF, keratinocyte growth factor (KGF), and TGF-α

Tissue was processed as described previously [21]. Using a microtome, six 4-μm-thick sections of check pouch tissue were obtained from each of the Normal, 5-FU/Saline, AZT1/5-FU, and AZT5/5-FU groups. Samples were transferred to gelatine-coated slides. Each tissue section was deparaffinised, rehydrated, washed with 0.3% Triton X-100 in phosphate buffer, and quenched with endogenous peroxidase (3% hydrogen peroxide). Tissue sections were incubated overnight at 4°C with primary antibodies (Santa Cruz Biotechnology, INTERPRISE, Brazil) against VEGF, FGF, KGF, and TGF-α (all at 1:400 dilution). Slices were washed with phosphate buffer and incubated with a streptavidin/horseradish peroxidase (HRP)-conjugated secondary antibody (Biocare Medical, Concord, CA, USA) for 30 min. Immunoreactivity to TGF, KGF, FGF, and VEGF was visualised by a colorimetric-based detection kit following the manufacturer’s protocol (TrekAvidin-HRP Label + Kit from Biocare Medical, Dako, USA).

### Myeloperoxidase (MPO) assay

The extent of neutrophil accumulation in Check pouch tissue samples was measured by assaying MPO activity. Cheek pouch mucosa (6 samples per group) was harvested as described above and stored at -70°C until required for assay. After homogenisation and centrifugation (2000 × *g* for 20 min), the MPO activity in these samples (in units of MPO/mg tissue) was determined by a previously described colorimetric method [22].

### Malonyldialdehyde (MDA) assay

Malonyldialdehyde (MDA) is an end product of lipid peroxidation. To quantify the increase in free radicals in check pouch tissue sample, MDA content was measured via the assay described by Esterbauer and Cheeseman [23]. Check pouch tissue samples (6 samples per group) were suspended in buffer Tris HCl 1:5 (w/v) and minced with scissors for 15 sec on an ice-cold plate. The resulting suspension was homogenised for 2 min with an automatic Potter homogenizer and centrifuged at 2500 × g at 4°C for 10 min. The supernatants were assayed to determine MDA content. The results are expressed as nanomoles of MDA per gram of tissue.

### IL-1β, IL-10 and TNF-α assay

Cheek pouch mucosa tissues (6 samples per group) were stored at -70°C until required for each assay. Collected tissue was homogenised and processed as described by [24]. Levels of IL-1β (detection range: 62.5–4000 pg/mL; sensibility or lower limit of detection [LLD]: 12.5 ng/mL of recombinant mouse IL-1β), IL-10 (detection range: 62.5–4000 pg/mL; sensibility or LLD: 12.5 ng/mL of recombinant mouse IL-10), and TNF-α (detection range: 62.5–4000 pg/mL; sensibility or LLD: 50 ng/mL of recombinant mouse TNF-α) in the samples were determined with a commercial ELISA kit (R&D Systems, Minneapolis, MN, USA), as described previously [25]. All samples were within the wavelength used in UV-VIS spectrophotometry (absorbance measured at 490 nm).

### Biochemical, white blood cell (WBC) and bacteraemia analyses

On day 10, animals were killed by an overdose of anaesthesia with 2% thiopental (80 mg/kg, i.p.). Blood samples were collected by heart puncture for subsequent biochemical, WBC, and bacteraemia analyses.

For biochemical analyses, serum was obtained by centrifuging total blood without anticoagulants at 2,500 rpm for 15 min. Serum levels of alanine amino transferase (ALT), aspartate amino transferase (AST), creatinine, and urea were determined by using standardised diagnostic kits (LABTEST) and spectrophotometry.

For WBC analysis, 20 μl of total blood were added to 380 μl of Turk solution. Total and differential counts of leukocytes (Number of leukocytes/mm³) were determined by standard manual procedures using light microscopy [26]. For bacteraemia analysis, 10 μl of total blood were diluted tenfold in brain-heart infusion media (BHI). Bacterial growth was analysed after 24 to 48 h at 37°C by visual analysis of the turbidity of the culture medium. A turbid medium indicates bacteraemia (+), and a non-turbid medium suggests absence of bacteria in the blood. Organisms that grew on BHI were streaked for isolation on 5% sheep blood agar plates, MacConkey agar plates, and mannitol-salt-agar plates. Organisms that grew were identified by standard methods, including Gram stain morphology, DNAse, catalase, coagulase, motility, and sugar metabolism methods.

### Statistical analysis

Data are presented as the mean ± standard error of the mean or as the median (range), when appropriate. Analysis of variance (ANOVA) followed by Bonferroni’s test was used to calculate the means. The Kruskal-Wallis test followed by Dunn’s test was used to compare medians (GraphPad Prism 5.0 Software, La Jolla, CA, USA). A p-value < 0.05 indicated a statistically significant difference. Analysis of MPO, MDA, IL-1β, IL-10 and TNF-α assay were performed in triplicate.

## Results

### Effects of AZT on OM

**Fig 1:**
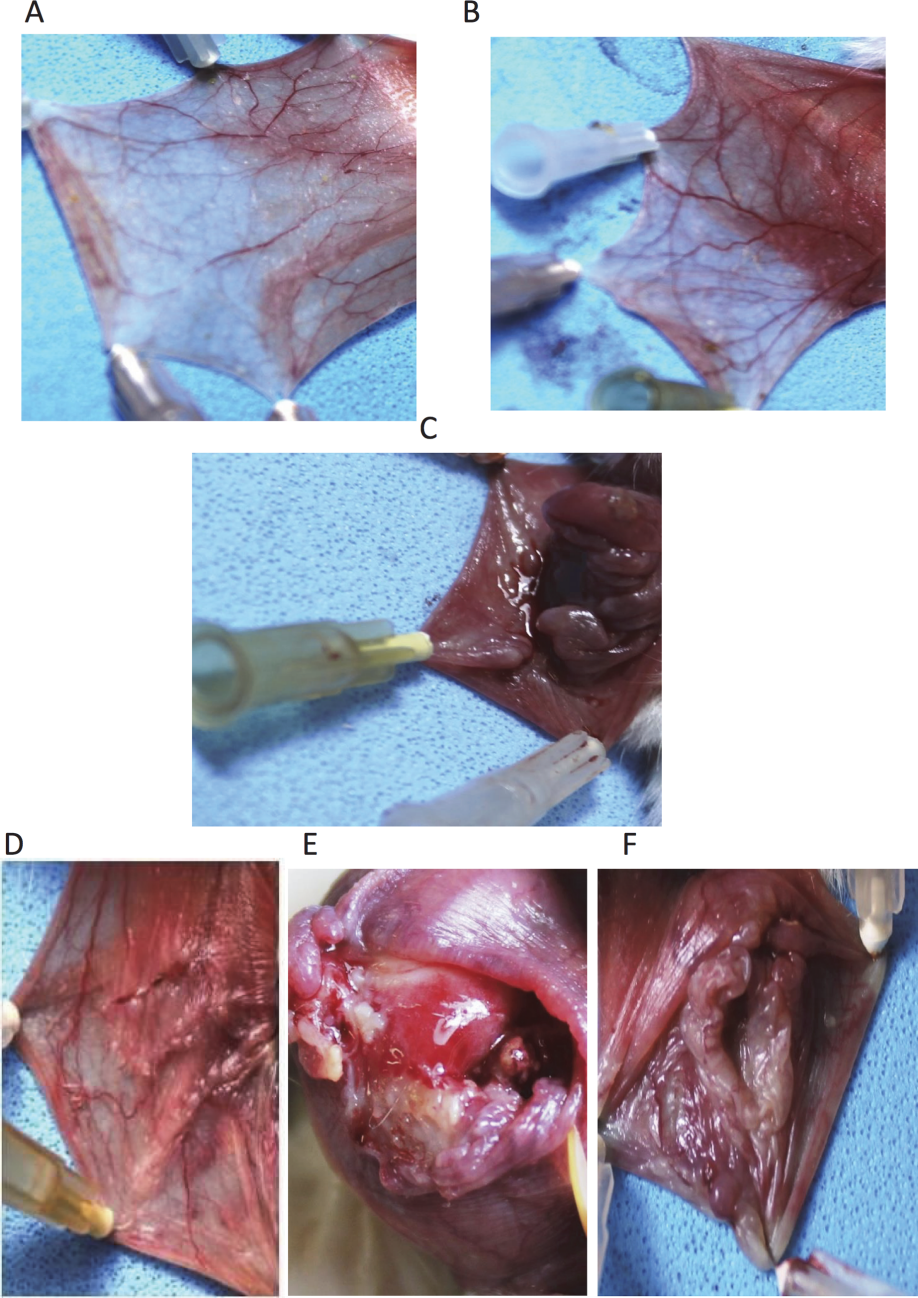
Macroscopic aspects of hamster cheek pouches. Representative cheek pouches are shown for animals subjected to: A: saline (Normal Group); B: mechanical trauma (MT Group); C: i.p. 5-FU on days 1 and 2 (60 mg/kg and 40 mg/kg, respectively), and MT of the cheek pouches on day 4 (5-FU/Saline Group); D: daily oral AZT (1 mg/kg), i.p. 5-FU on days 1 and 2 (60 mg/kg and 40 mg/kg, respectively), and MT of the cheek pouches on day 4 (AZT1/5-FU Group); E: daily oral AZT (5 mg/kg), i.p. 5-FU on days 1 and 2 (60 mg/kg and 40 mg/kg, respectively), and MT of the cheek pouches on day 4 (AZT5/5-FU Group); F: daily oral AZT (10 mg/kg), i.p. 5-FU on days 1 and 2 (60 mg/kg and 40 mg/kg, respectively), and MT of the cheek pouches on day 4 (AZT10/5-FU Group).

**Fig 2:**
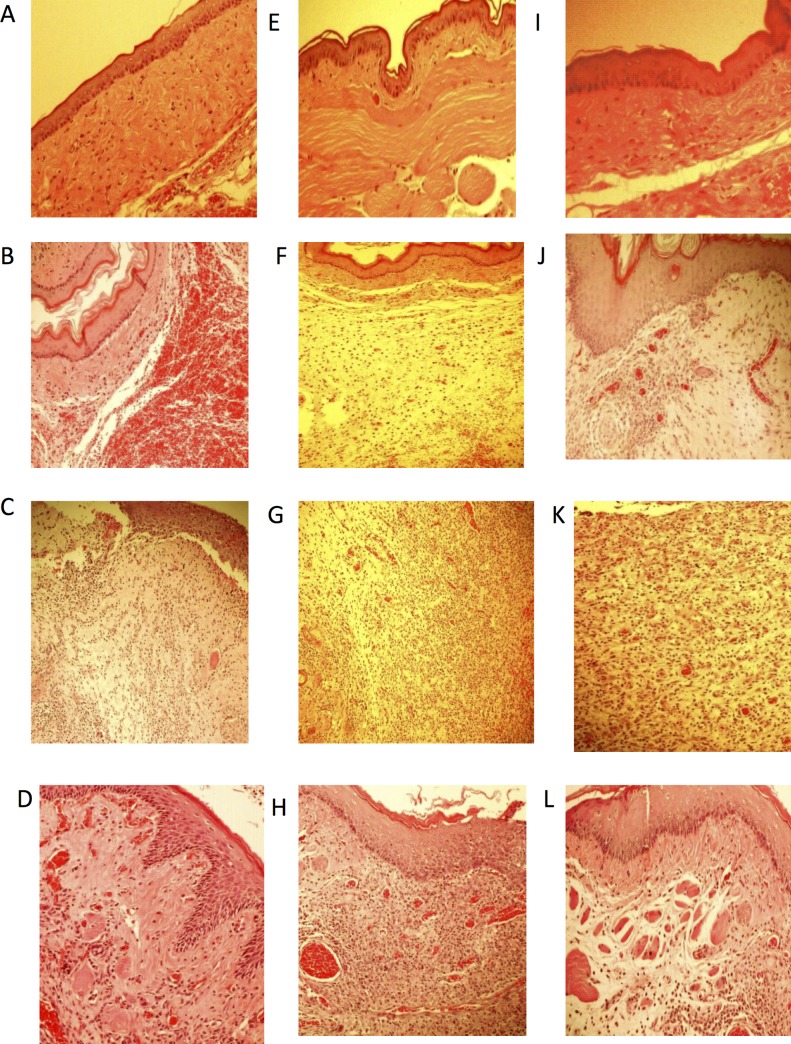
Microscopic aspects of hamsters cheek pouches. Animals subjected to without oral mucositis that received saline (Normal) (A, E, I); Animals subjected to Trauma in oral mucosa that received Saline (MT) (B, F, J); Animals subjected to 5-FU/saline that received Saline (C, G, K); Animals subjected that received AZT1/5-FU (D). Animals subjected that received AZT5/5-FU (H), Animals subjected that received AZT10/5-FU.

Treatment of hamsters with 5-FU followed by MT of the cheek pouch (5-FU/Saline group) caused lesions within 10 days, as evidenced by erythema, hyperaemia, haemorrhagic areas, and extensive ulceration. Clinical dates were confirmed by histopathologic analysis, which revealed severe vascular ingurgitation and vasodilatation, accentuated inflammatory infiltration with the prevalence of neutrophils, oedema, and extensive ulceration and abscesses (Figs. 1 and 2, Table 1). On day 10, the median (range) for the macroscopic score was 5 (4.5–5) and for the histologic score was 4 (4–4).

**Table 1:** Macroscopic, microscopic, Number of leucocytes/mm^3^ and N° With Bacteremia/Total(Microorganism Identification) analysis of hamsters check pouches subjected of experimental oral mucositis, Natal, RN, 2014.

Groups	Macroscopic Analysis	Microscopic Analysis	Number of leukocytes/mm³	N° With Bacteremia/Total (Microorganism Identification)
Normal	0 (0–0)###	1(1–1) ###	5483 ± 1412	0/6
MT	2 (1.5–3)#	2(2–2) ##	4260 ± 2569	2/5 (no determinate)
5-FU/Saline	5 (4.5–5)	4(4–4)	25000 ± 11174 ***	4/6 (+) Staphylococcus xilosus
AZT 1/5-FU	2 (2–3.5) #	2 (2–2) ##	8300± 4622 ##	0/4
AZT5/5-FU	4 (4–4)	3 (2.25–3)	13660 ± 8065	2/5 (+) Pastereulla caninis Staphylococcus aureus
AZT10/5-FU	3 (3–3)	3 (3–3.75)	9540 ± 3777 ##	4/5(+) Enterobacter cloacae, Aerococcus viridans, Staphylococcus auricularis, Proteus mirabilis

(#p <0.05, ##p < 0.01, ###p<0.001 compared groups with 5-FU/saline; ***p<0.001 compared groups with Normal Group)

Compared to the 5-FU/Saline group, the MT group evidenced less severe clinical lesions, with discrete erythema but no ulcers. Clinical dates were confirmed by histopathologic analysis, which showed reduced levels of cellular inflammation, oedema, and haemorrhage, although granulation tissue was still observed (Figs. 1 and 2, Table 1). On day 10, the median (range) for the macroscopic score was 2 (1.5–3) and for the histologic score was 2 (2–2) (^#^ p< 0.5 and ^##^p < 0.01 vs. 5-FU/Saline group).

Compared to the 5-FU/Saline group, the AZT1/5-FU group showed less severe clinical lesions, with discrete erythema but no ulcers. Histopathologic analysis revealed reduced cellular inflammation, oedema, and haemorrhage, but granulation tissue was still present (Figs. 1 and 2, Table 1). On day 10, the median (range) for the macroscopic score was 2 (2–3.5) and for the histologic score was 2 (2–2) (^#^ p < 0.5 and ^##^p< 0.01 vs. 5-FU/Saline group).

**Fig 3:**
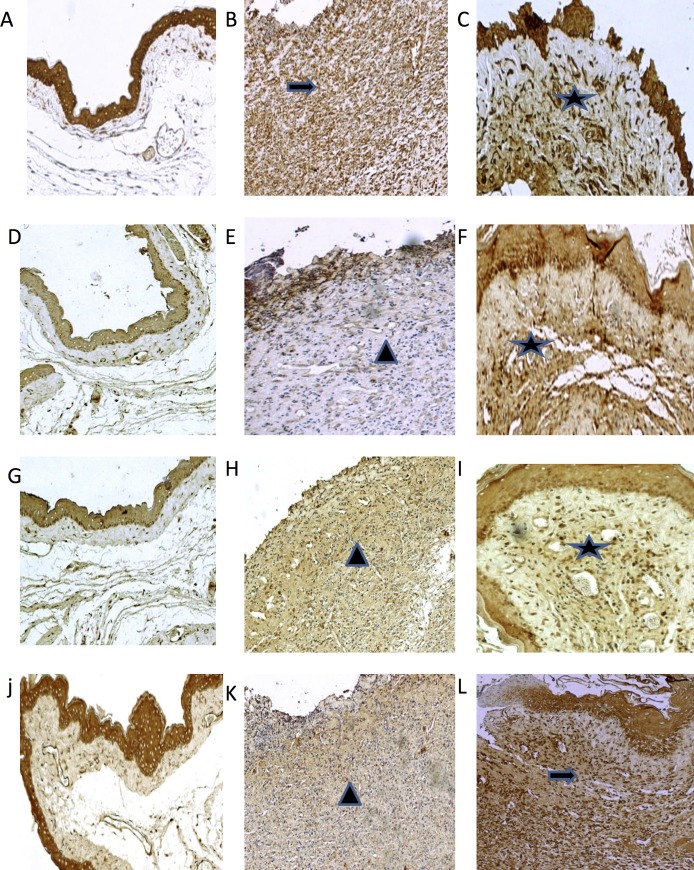
Photomicrographs of hamsters cheek pouches showing immunoreactivity to TGF-α, FGF, KGF and VEGF. Hamsters subjected to Normal (A, D, G, J, M, P, S); Hamsters subjected to 5- FU/saline (B, E, H, K, N, Q, T); Hamsters subjected to treated with AZT1/5-FU (C, F, I, L, O, R, U) and Hamsters subjected to treated with AZT5/5-FU (C, F, I, L, O, R, U). Images are shown at 40× magnification. Bar = 100 μm. Arrow indicates high or moderate labeling in the oral mucosa. Asterisk indicates mild or moderate labeling in the oral mucosa. Triangle and asterisk indicate oral mucosa. Triangle and arrow indicate high labeling of oral mucosa. Asterisk and triangle indicate mild labeling of oral mucosa.

Compared to the 5-FU/Saline group, the AZT5/5FU and AZT10/5FU groups did not evidence significant reductions in the severity of the clinical lesions. These groups presented erythema, hyperaemia, haemorrhagic areas, and extensive ulceration. Histopathologic analysis showed cellular inflammation, with prevalence of neutrophils, oedema, ulcers, and abscesses (Figs. 1 and 2, Table 1). Medians (ranges) for the macroscopic scores were 4 (4–4) and 3 (3–3), respectively, and for the histologic scores were 3 (2.25–3) and 2 (3–3.75), respectively, on day 10 (all p > 0.05 vs. 5-FU/Saline group). Differences in immunohistochemistry markers on day 10 were observed among the Normal, 5-FU/Saline, AZT1/5-FU, and AZT5/5-FU groups (Fig. 3). Treatment with AZT at 1 mg/kg (AZT1/5FU) caused a considerable increase in immunostaining for VEGF, FGF, KGF, and TGF-α in the check pouch tissues when compared to animals in the 5-FU/Saline group.

### MPO and MDA levels

**Fig 4:**
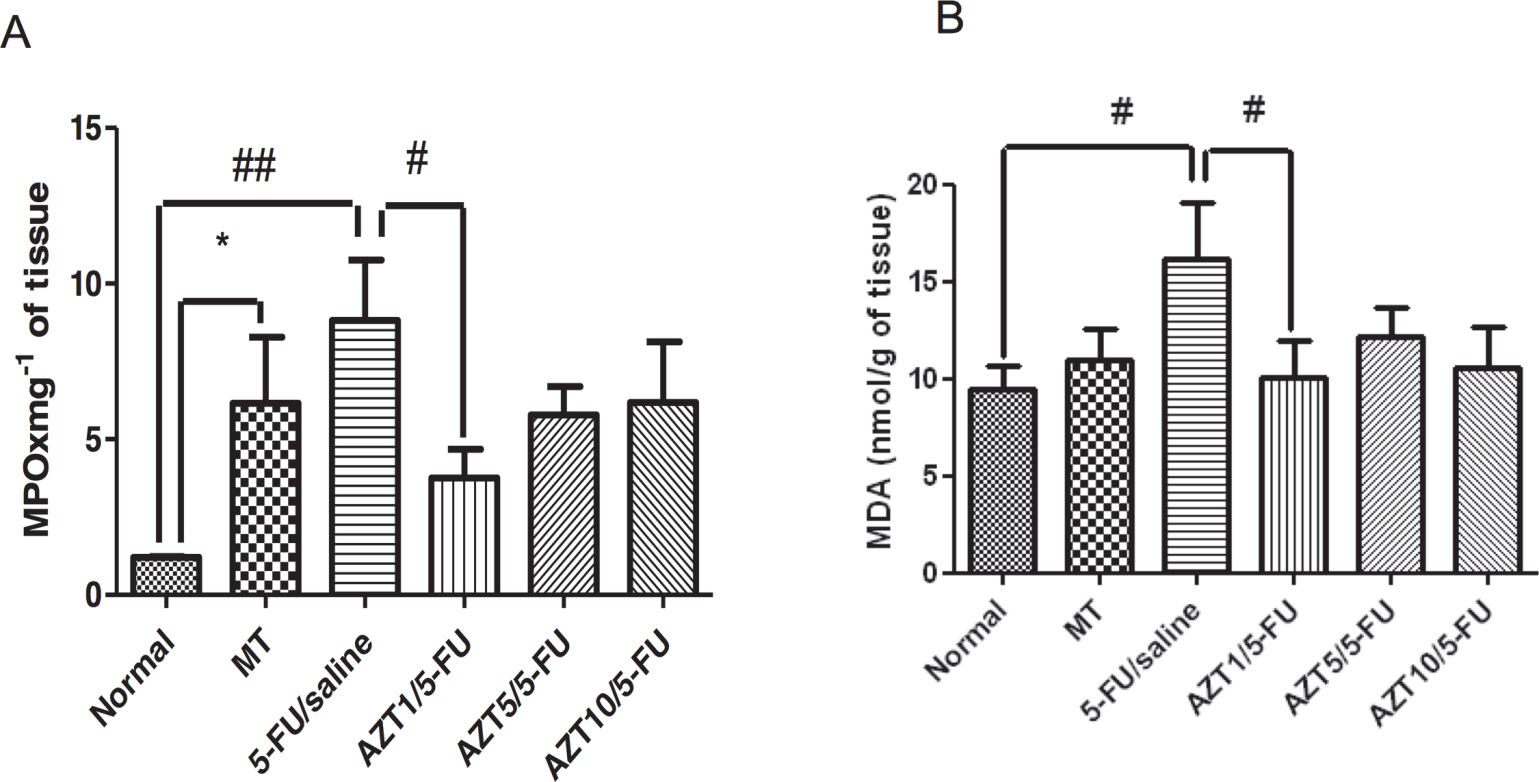
MPO (A) and MDA (B) in Normal, MT, 5-FU/saline and groups treated with AZT1/5-FU, AZT5/5-FU and AZT10/5-FU (#*p* <0.05, ##*p* < 0.01, compared groups with 5-FUT/saline Group; *p<0.05, compared groups with Normal Group).

The MPO activity was measured in the cheek pouch as an indicator of neutrophil infiltration. Levels of MPO in the Normal and AZT1/5-FU groups were decreased (p <0.01 and p < 0.05, respectively) compared to levels in the 5-FU/Saline group (Fig. 4A). Levels of MPO in the MT group were increased (p < 0.05) compared to levels in the Normal group (Fig. 4A). Levels of MDA in the Normal and AZT1/5-FU groups were decreased (*p* < 0.05) compared to levels in the 5-FU/Saline group (Fig. 4B).

### IL-1β, IL-10, and TNF-α levels

**Fig 5:**
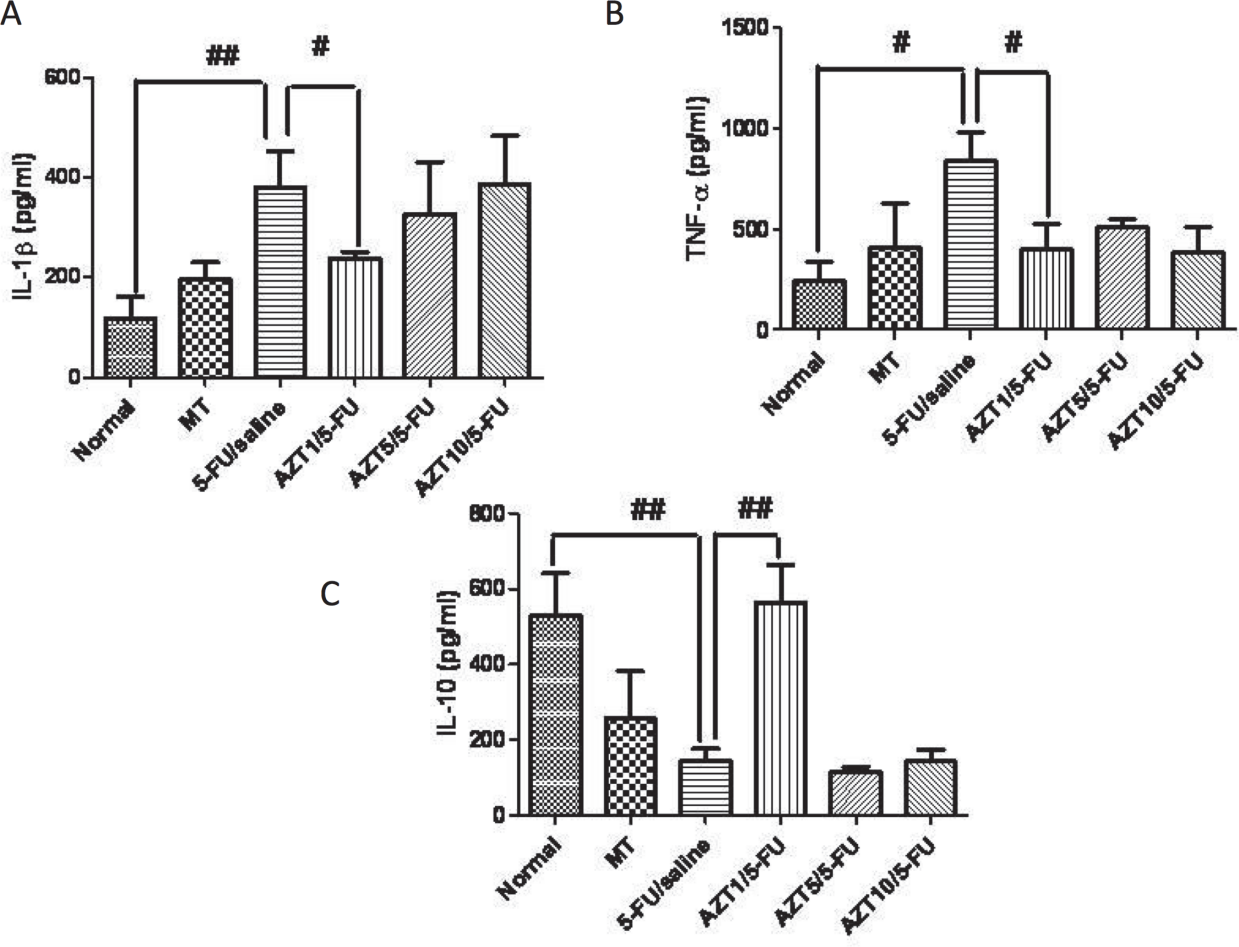
Levels of IL-1β (A), TNF- α (B) and IL-10 (C), and, Normal, MT, 5-FU/saline and groups AZT1/5-FU, AZT5/5-FU and AZT10/5-FU (#*p* <0.05, ##*p* < 0.01, compared group with 5-FU/saline).

Levels of IL-1β and TNF-α in the Normal group were decreased compared to levels in the 5-FU/Saline group (p <0.01, Fig. 4A and p < 0.05, Fig. 4B, respectively). Levels of IL-10 in the Normal group were decreased compared to levels in the 5-FU/Saline group (p <0.01, Fig. 4C). Treatment with AZT at 1 mg/kg (AZT1/5-FU) significantly blocked this elevation in TNF-α (p<0.05) and IL-1β (p<0.05) levels in the cheek pouch (Fig. 5A and 5B, respectively), while significantly increasing the levels of anti-inflammatory cytokine IL-10 compared to the 5-FU/Saline group (p < 0.01, Fig. 5C).

### Effects of AZT on systemic changes: Biochemical, WBC and bacteraemia analyses

To determine the effect of AZT on systemic changes, biochemical, WBC, and bacteraemia analyses were performed. Levels of hepatic enzymes (ALT and AST) and markers of renal function (urea and creatinine) did not show a significant difference between the groups (Table 2). On day 10, animals in the 5-FU/Saline group presented significantly increased leukocyte levels compared to the Normal control group (p < 0.001; Table 1). Table 1 shows the results of the bacteraemia analysis for animals in the various groups on day 10. Animals in the Normal control group did not exhibit bacteraemia (-). The 5-FU/Saline group had the highest proportion of animals with bacteraemia (*Staphylococcus xylosus* was found in 4/6 samples). The AZT1/5-FU group did not present any sample with bacteraemia. Bacteraemia (+) samples were found in the AZT5/5-FU and AZT10/5-FU groups, and different bacterial species were identified.

**Table 2:** Determination of the alanine amino transferase (ALT) and aspartate amino transferase (AST), creatinine and urea parameters Biochemistry of experimental oral mucositis, Natal, RN, 2014.

Groups	ALT	AST	Urea	Creatinine
Normal	92.8 ± 4.9	56.2 ± 19.3	47. 4 ± 7.7	0.2 ± 0.08
MT	92.0 ± 17.1	44.4 ± 16.7	50.8 ± 15.9	0.18 ± 0.08
5-FU/Saline	115 ± 49.4	78.5 ± 58.7	38.9 ± 10.0	0.28 ±0.1
AZT 1/5-FU	87.3 ± 22.6	50.5 ± 12.7	42 ± 4.8	0.3 ± 0.0
AZT5/5-FU	68.4 ± 13.1	57 ± 37.7	38 ± 5.6	0.27 ± 0.5
AZT10/5-FU	86.8 ± 15.9	45 ± 12.4	37 ± 5.6	0.25 ± 0.05

## Discussion

AZT is an ARB that was approved for use in 2011. At its maximal dose, AZT has superior efficacy to both olmesartan and valsartan at their maximal, approved doses, without increasing adverse events [27]. Studies have shown that AZT exhibits anti-inflammatory activity [18], confirming reports regarding the anti-inflammatory activities of ARBs [17,28,29].

The 5-FU/Saline group in this study showed a median macroscopic score of 5, with nearly complete ulceration of the cheek pouch mucosa and fibrosis that made exposure of the oral mucosa difficult. These findings differed significantly from those of the AZT1/5-FU group (median macroscopic score = 2), which evidenced erythema and vasodilation but no ulceration. The results were confirmed by histopathologic analysis, which revealed reduced infiltration of inflammatory cells and oedema. Treatment of animals with 5 mg/kg AZT (AZT5/5-FU group) or 10 mg/kg AZT (AZT10/5-FU group) did not reduce the 5-FU–induced lesions in the cheek pouch, as indicated by the macroscopic and histologic scores.

The histopathologic results confirmed the presence of granulation tissue in the AZT1/5-FU group. The macrophages in granulation tissue control the cellularity of wounds by inducing apoptosis and phagocytising various wound cells. Macrophages target neutrophils during the inflammatory phase of repair, as well as fibroblasts and endothelial cells (ECs) during the resolution of this phase [30]. Neutrophils become less prevalent as the wound matures, and macrophages emerge as the predominant inflammatory cell. Neutrophils undergo apoptosis in the wound and are recognised and ingested by macrophages [31]. We found that the AZT1/5-FU group showed a significant reduction in the number of neutrophils, as confirmed by the significant reduction of level MPO.

Wound healing is an evolutionarily conserved, complex, multicellular process that, in skin, aims at barrier restoration. This process involves the coordinated efforts of several cell types, including keratinocytes, fibroblasts, ECs, macrophages, and platelets. The migration, infiltration, proliferation, and differentiation of these cells culminate in inflammation, new tissue formation, and, ultimately, wound closure. This complex process is executed and regulated by an equally complex signalling network involving numerous growth factors, cytokines, and chemokines [32].

OM is characterised by an intense inflammatory reaction, caused by the effects of chemotherapeutic agents on the mucosa lamina propria cells, which results in the production of proinflammatory cytokines (e.g., IL-1β, IL-6, and TNFα) [33]. We demonstrated that oral treatment with 1 mg/kg AZT significantly (*P* < 0.05) reduced TNF-α and IL-1β levels in an experimental model of OM in hamsters. In agreement with our data, various articles have shown that AZT decreases IL-1β production [18].

IL-10 is the important cytokine with anti-inflammatory properties. In monocytes and macrophages, IL-10 diminishes the production of inflammatory mediators and inhibits antigen presentation, although it enhances their uptake of antigens [34]. In our study, the AZT1/5-FU group showed significantly increased levels of IL-10 and TGF-α compared to 5FU/saline group. Increased tissue levels of TGF-α can indicate macrophage migration to granulation tissue [35]. Macrophages initiate granulation tissue development and release various growth factors, including FGF, EGF, TGF, and platelet-derived growth factor (PDGF) [32]. Macrophages provide an ongoing source of cytokines to modulate inflammatory cell adhesion, cell migration, and fibroblast proliferation. Within hours of injury, re-epithelialisation is initiated, and the release of EGF, TGF-α, and FGF acts to stimulate epithelial cell migration and proliferation [36].

TGF-α has the ability to increase keratinocyte migration and proliferation [14]. FGFs are produced by keratinocytes, fibroblasts, ECs, smooth muscle cells, chondrocytes, and mast cells. FGF-2 or FGF is increased in the acute wound and plays a role in granulation tissue remodelling [16]. FGF-7 or KGF-1, which may be important during the late stages of neovascularisation, upregulates VEGF [37], stimulates the proliferation and migration of keratinocytes, and plays an important role in re-epithelialisation [36]. In the present investigation, the AZT1/5-FU group evidenced increased tissue expression of growth factors (i.e., VEGF, FGF, KGF, and TGF-α) involved in the healing process and, especially, in the activation of keratinocytes and neovascularisation with collagen scaffold formation.

The use of AZT was tested in three doses; however, only the 1 mg/kg dose (AZT1/5-FU group) showed efficacy. At this dose, the inflammatory process was reduced, with a significant reduction in MPO levels, notable presence of granulation tissue, and increased levels of IL-10, VEGF, FGF, KGF, and TGF-α. The use of AZT showed a dose-independent effect, with preference for a lower dose in order to achieve the best clinical results by accelerating the healing process. The effect of AZT on systemic changes was tested through biochemical, WBC, and bacteraemia analyses. AZT proved to be a very safe drug; even associated with 5-FU (used to induce OM), AZT did not cause changes in the biochemical parameters. With the WBC and bacteraemia analyses, bacteraemia was found in four of six samples in the 5-FU/Saline group, which also showed significant leukocytosis. Although 5-FU treatment is expected to result in leukopenia, the observed leukocytosis was probably in response to bacterial lipopolysaccharide or host proinflammatory cytokines as a result of secondary infection. The AZT1/5-FU group no showed the presence of bacteria in samples. These findings strengthen the evidence for a protective role of AZT in 5-FU–induced OM.

A limitation of the present study is the use of an experimental model of oral mucositis that was established in hamsters. Given that major genetic differences exist between rodents and humans, the present results need to be confirmed in primates prior to the clinical application of azilsartan to humans.

In the present study, we show that azilsartan mediates a protective effect towards the inflammatory parameters that characterize 5-FU-induced OM. Many of the functional effects demonstrated are dependent on two key factors related to azilsartan as a molecule: 1) it has a high affinity and slow dissociation rate from AT1R, and 2) it exhibits inverse agonistic properties. These properties suggest that azilsartan represents a unique therapeutic agent for the potential treatment of a wide range of angiotensin II-dependent cardiometabolic diseases. These diseases include cardiac hypertrophy, unstable atherosclerotic plaques, cardiac fibrosis, insulin resistance, renoprotection and antiinflammatory effects [38,39]. In addition, the results obtained from a number of clinical studies indicate that angiotensin II (AII) receptor type 1 (ATR1)-blocking drugs (ARBs) mediate anti-inflammatory effects [40–42].

In conclusion, the findings in this study demonstrate that AZT accelerates the healing process in an experimental model of 5-FU–induced OM, stimulating granulation tissue formation, fibroblast and keratinocyte migration, and collagen deposition. These data were confirmed by the increased levels of TGF-α, FGF, and KGF, as well as the upregulation of VEGF, which has been shown to promote angiogenesis in scar tissue. Consequently, use of AZT at a dose of 1 mg/kg accelerates the healing process by activating growth factors involved in angiogenesis and re-epithelialisation, in addition to increasing levels of the anti-inflammatory cytokine IL-10. Application of azilsartan-associated chemotherapy to the model of oral mucositis that was established did not induce an anti-inflammatory effect. It remains to be determined whether hypertensive cancer patients will benefit from an anti-inflammatory effect mediated by azilsartan-based chemotherapy
